# Supplementary figures and images for: Mathematical modeling of the evolution of resistance and aggressiveness of high-grade serous ovarian cancer from patient CA-125 time series
Source: PLoS Comput Biol. 2024 May 29;20(5):e1012073. doi: 10.1371/journal.pcbi.1012073 (PMC11164342; doi:10.1371/journal.pcbi.1012073)

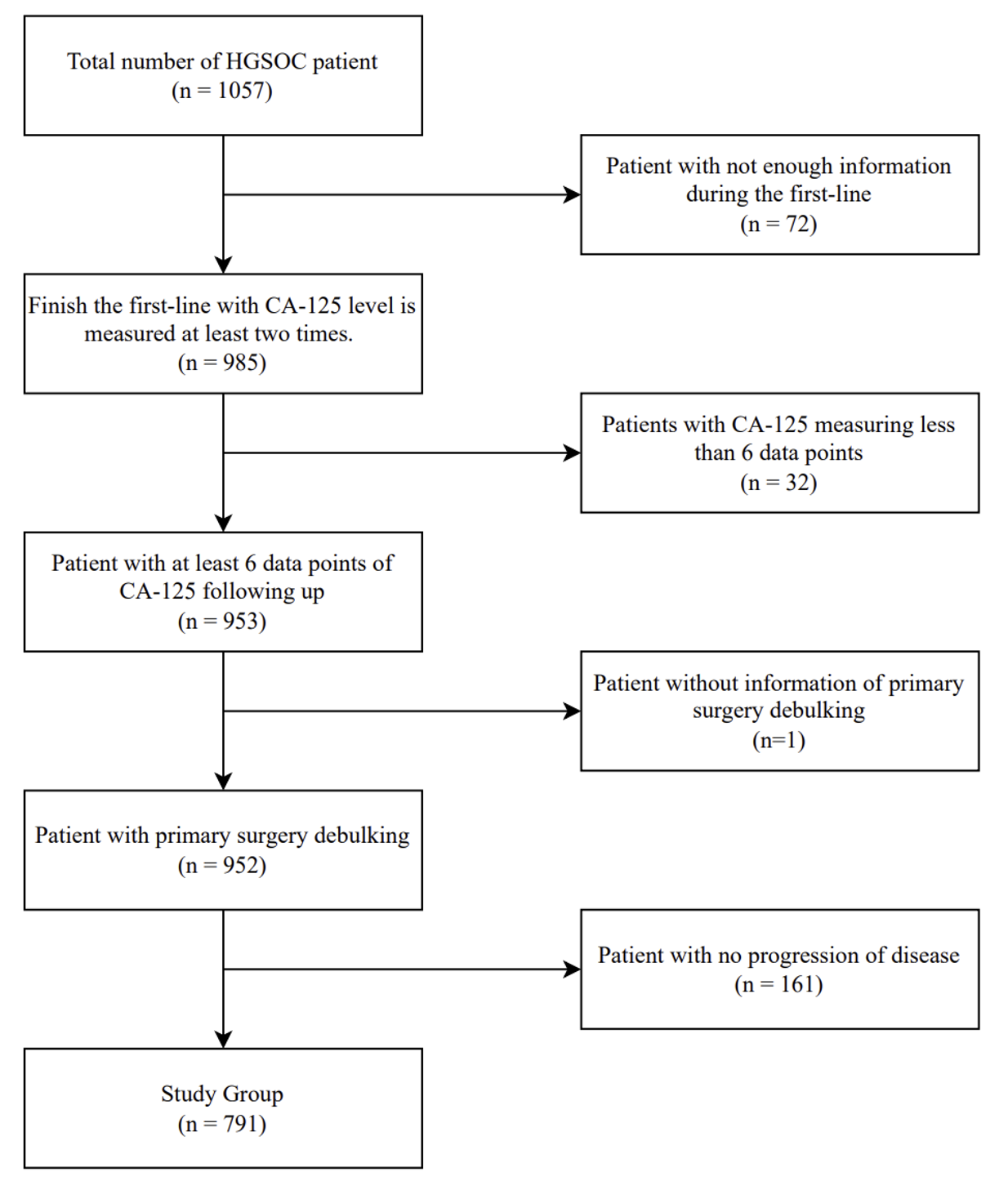

Supplement: S1 Fig — The qualification of patients in the study. The diagram summarizes the inclusion and exclusion criteria in the study. (TIF) [file pcbi.1012073.s001.tif]

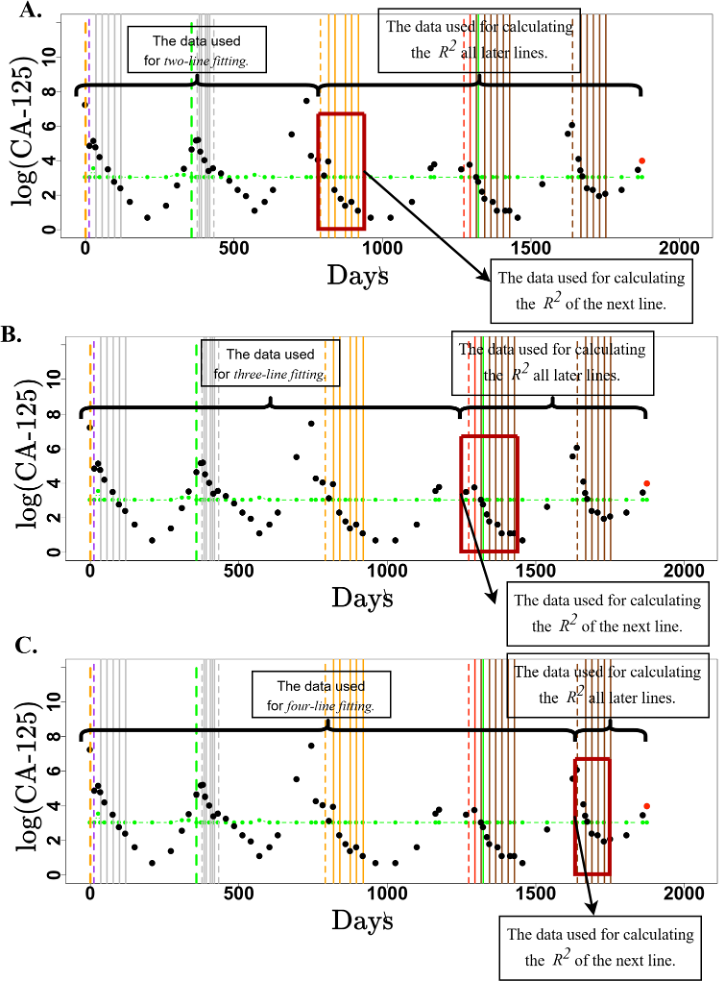

Supplement: S2 Fig — Scatter plot of log-transformed CA-125 level with time (days). A graphical representation of the data used for the model fitting of A. two-lines fitting B. three-lines fitting C. four-lines fitting. Data utilized in calculating the R2 value for all unfitted lines (R2 all later lines) and the R2 value for the subsequent line (R2 the next line). Each individual vertical line denotes the date on which the patient received treatment doses, which is Cyle of treatment. The group of cycles together with gap between them is Line of treatment. The different colors of the vertical lines indicate the use of distinct drugs in each treatment line. The first-line, second-line, third-line, fourth, and fifth-line indicate the line of treatment. The indicator variable is α = 1 during lines of treatment and α = 0 otherwise. (TIF) [file pcbi.1012073.s002.tif]

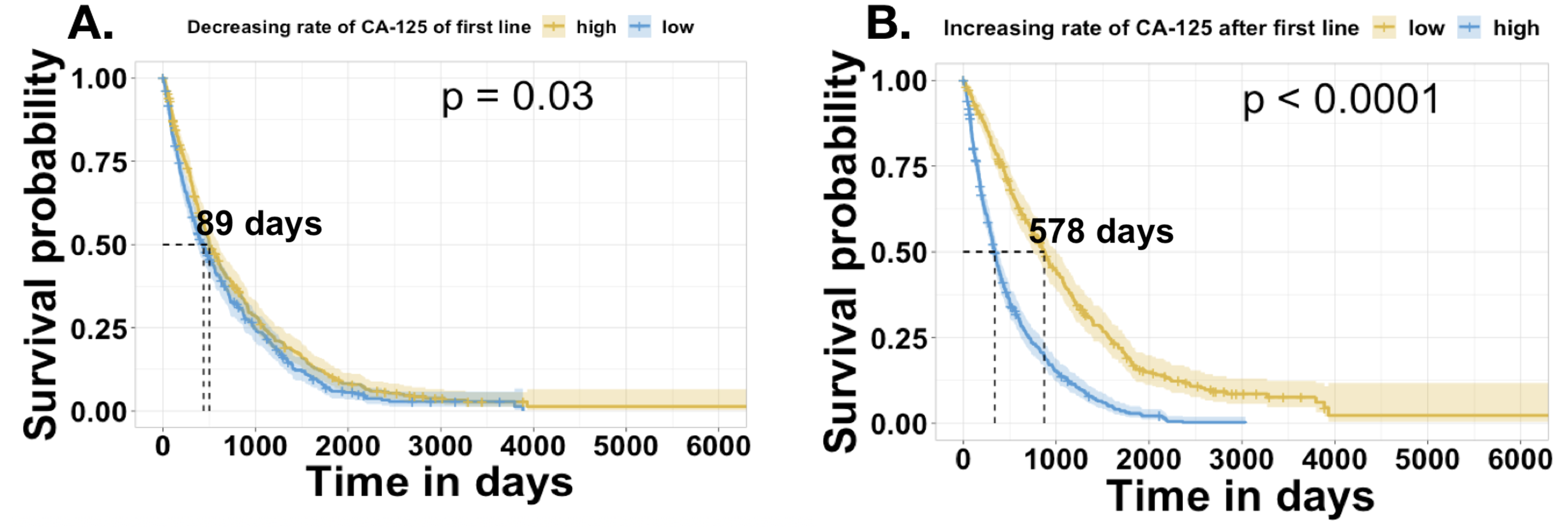

Supplement: S3 Fig — Kaplan-Meier plots of survival after finishing the second line of treatment. A. Effect of data-based resistance during first-line treatment. B. Effect of data-based aggressiveness after first-line treatment. (TIF) [file pcbi.1012073.s003.tif]

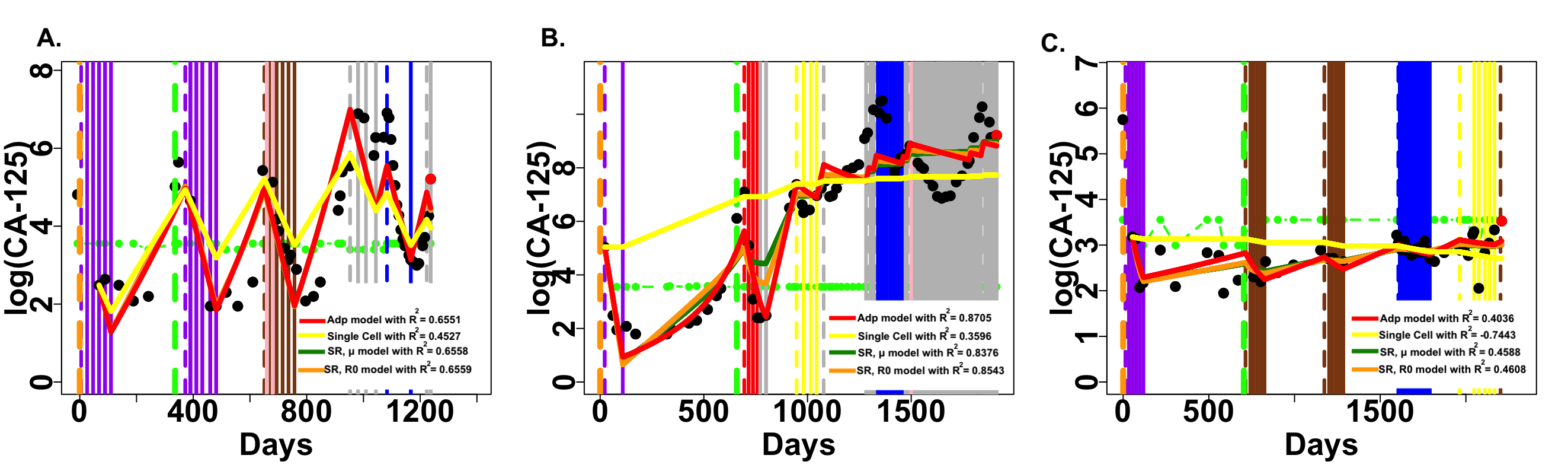

Supplement: S4 Fig — Examples of patients with the three patterns of CA-125 dynamics. The lines illustrate model fits using all data points for the three models: the SR and R0 model (orange), the SR and μ model (green), the single-cell model (yellow), and the adaptive dynamics model (red). (TIFF) [file pcbi.1012073.s004.tiff]

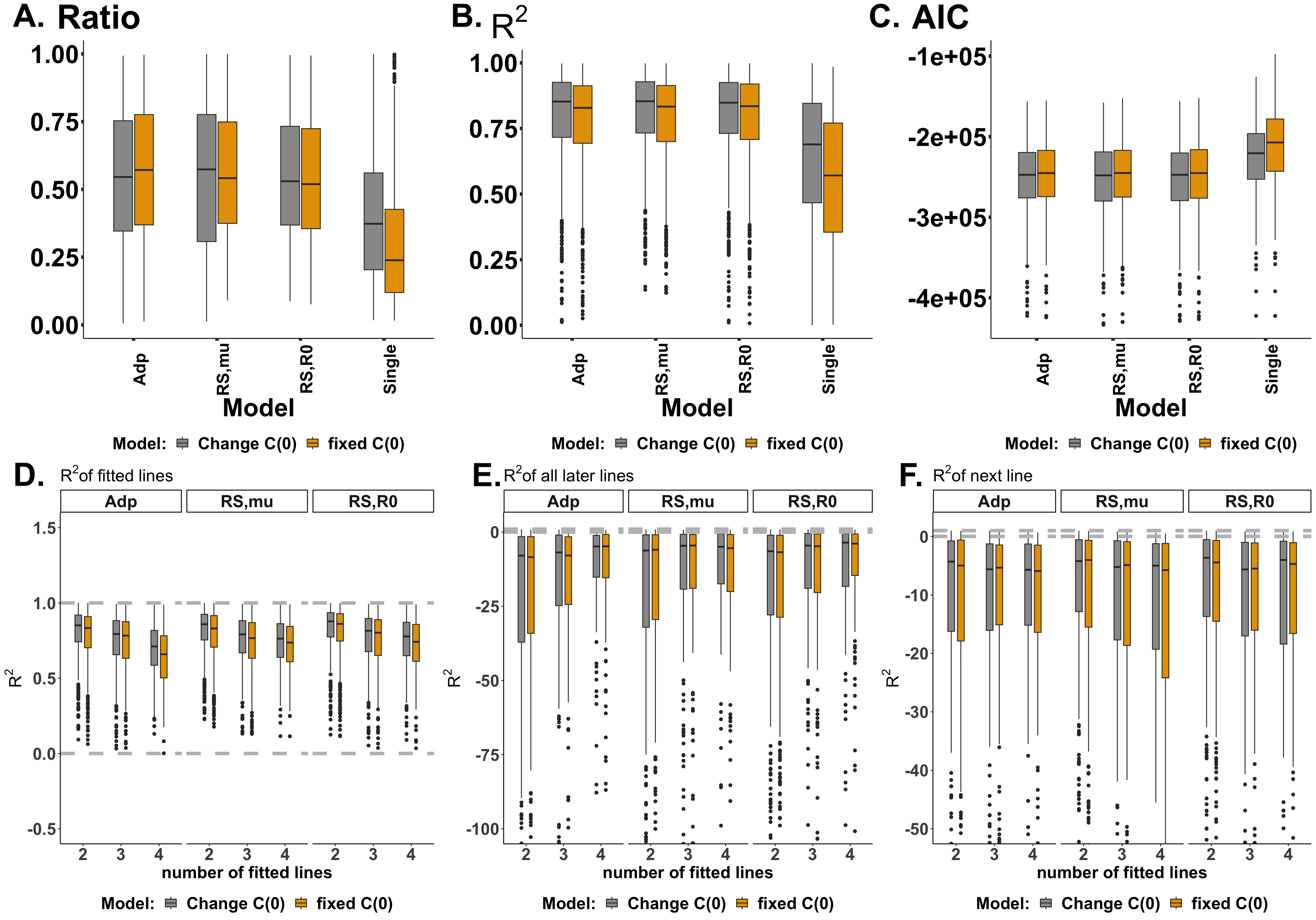

Supplement: S5 Fig — A. Box plot of the ratio of the sum of the squared errors from the Friedman’s SuperSmoother function to the sum of the squared errors from the mathematical models. B. Box plot of goodness of fit (R2) using all-lines fitting. C. The Akaike Information Criterion (AIC) of the four models. The red dot indicates the median value of each variables. Box plot showing R2 of predictions of D. the data used for model fitting E. the next line after the last fitted line and F. all the lines after the last fitted line using two-line fitting, three-line fitting, and four-line fitting. (TIFF) [file pcbi.1012073.s005.tiff]

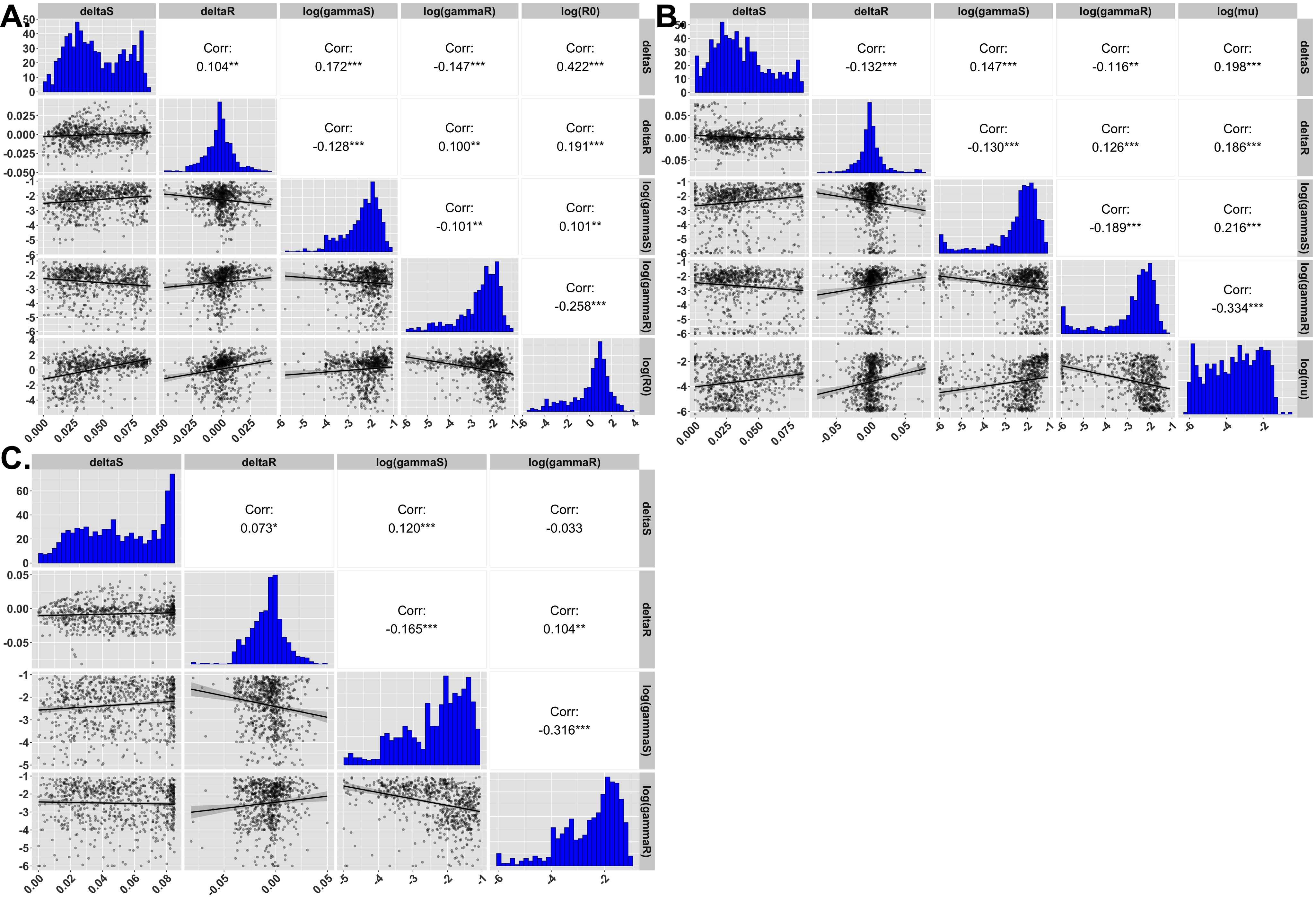

Supplement: S6 Fig — Pairwise correlation comparison of estimated parameters from mathematical models using all-line fitting with the scatter plots, histogram representing distribution, and the correlation coefficient A. the SR and R0 model B. the SR and μ model, and C. the Adp model. (TIFF) [file pcbi.1012073.s006.tiff]
