# Supplementary material for: Mathematical modeling of the evolution of resistance and aggressiveness of high-grade serous ovarian cancer from patient CA-125 time series
Source: PLoS Comput Biol. 2024 May 29;20(5):e1012073. doi: 10.1371/journal.pcbi.1012073 (PMC11164342; doi:10.1371/journal.pcbi.1012073)
Supplement: S1 Table — Summary and correlations of the data-based resistance estimated from the first four lines of therapy. Negative values indicate a decrease in CA-125, and positive values show an increase in CA-125. The log(CA-125) in the last column is the level of CA-125 before the patients are given therapy. Italics indicates significance at p < 0.05 and bold p < 0.0001. (PDF) [file pcbi.1012073.s007.pdf]

	Resistance line 1	*Resistance* line 2	*Resistance* line 3	*Resistance* line 4	log(CA-125)
Mean	-0.0235	-0.0129	-0.0084	-0.0055	6.305
s.d.	0.0095	0.0096	0.0099	0.0113	1.686
range	(-0.0607,0.0057)	(-0.0601,0.0318)	(-0.0636,0.0203)	(-0.0557,0.0271)	(1.099,11.440)
Resistance line 1	-	*0.1093*	*0.0912*	0.0443	**-0.4984**
Resistance line 2		-	0.0782	0.0400	0.0591
Resistance line 3			-	**0.2069**	-0.0190
Resistance line 4				-	-0.0557
