# Supplementary material for: Mathematical modeling of the evolution of resistance and aggressiveness of high-grade serous ovarian cancer from patient CA-125 time series
Source: PLoS Comput Biol. 2024 May 29;20(5):e1012073. doi: 10.1371/journal.pcbi.1012073 (PMC11164342; doi:10.1371/journal.pcbi.1012073)
Supplement: S2 Table — Summary and correlations of the data-based aggressiveness estimated from the first four lines of therapy. Italics indicates significance at p < 0.05 and bold p < 0.0001. (PDF) [file pcbi.1012073.s008.pdf]

	Aggressiveness line 1	*Aggressiveness* line 2	*Aggressiveness* line 3	*Aggressiveness* line 4
Mean	0.00857	0.0099	0.0101	0.0075
s.d.	0.0075	0.00603	0.0055	0.0047
range	(0.0017,0.0445)	(0.0070,0.0384)	(0.0080,0.0203)	(0.0051,0.0262)
Aggressiveness line 1	-	**0.2858**	0.0662	*-0.1*
Aggressiveness line 2		-	*0.2466*	0.0067
Aggressiveness line 3			-	*0.1380*
