# Supplementary material for: Mathematical modeling of the evolution of resistance and aggressiveness of high-grade serous ovarian cancer from patient CA-125 time series
Source: PLoS Comput Biol. 2024 May 29;20(5):e1012073. doi: 10.1371/journal.pcbi.1012073 (PMC11164342; doi:10.1371/journal.pcbi.1012073)
Supplement: S1 File — Additional results that are not present in the main paper include the fitting from the Adaptive Dynamic model and the SR model with μ. (PDF) [file pcbi.1012073.s009.pdf]

Supporting information.

Mathematical Model

Sensitive and resistant cell model (SR model)

The SR and R0 model

In this model, treatment-resistant cells present before the administration of first-line therapy, and treatment-sensitive cells do not transform into treatment-resistant cells during treatment ($\mu = 0$). The model can be rewritten as treatment:

During Chemotherapy treatment:

$$\frac{dR}{dt} = -\delta_R R$$
$$\frac{dS}{dt} = -\delta_S S,$$

Off Chemotherapy treatment:

$$\frac{dR}{dt} = \gamma_R R$$
$$\frac{dS}{dt} = \gamma_S S,$$

where $C(t) = S(t) + R(t)$ and $R(0) > 0$.

The SR and μ model

In this model, treatment-resistant cells are not present prior to the initiation of chemotherapy but arise from the transformation of treatment-sensitive cells. ($\mu > 0, R(0) = 0$). The equations are

During Chemotherapy treatment:

$$\frac{dR}{dt} = -\delta_R R + \mu S$$
$$\frac{dS}{dt} = -\delta_S S - \mu S,$$

Off Chemotherapy treatment:

$$\frac{dR}{dt} = \gamma_R R + \mu S$$
$$\frac{dS}{dt} = \gamma_S S - \mu S,$$

with the initial condition $S(0) = C(0)$ and $R(0) = 0$.

The single-cell type model.

The model assumes that the patients do not change chemoresistance throughout the course of treatment ($\mu = 0, R(0) = 0$). The model is given by

<u>During Chemotherapy treatment:</u>

$$\frac{dS}{dt} = -\delta_S S,$$

<u>Off Chemotherapy treatment:</u>

$$\frac{dS}{dt} = \gamma_S S,$$

where $C(t) = S(t)$.

Data inclusion in the study

This study includes 791 HGSOC patients for mathematical modeling data fitting and statistical analysis. For each mathematical model, the parameters were estimated using four different numbers of lines of treatment in the fitting:

- all the lines of treatment (*all-line fitting*) (n = 791 patients)
- The first two lines of treatment (*two-line fitting*): We use the patients who finished at least three lines of chemotherapy (n = 545 patients)
- The first three lines of treatment (*three-line fitting*): We use the patients who finished at least four lines of chemotherapy (n = 343 patients)
- The first four lines of treatment (*four-line fitting*): We use the patients who finished at least five lines of chemotherapy (n = 210 patients).

Statistical results

Characteristic		Median value or number [range or %]
Total number of patients		791
Age (years)		60.24 [53.83,67.3]
CA-125 before treatment start		704 [261.5, 1835.0]
PFI(days)		321.0 [189, 592.5]
overall survival after the second line (days)		440.0 [180, 924]
Residual disease	≤ 1 cm	185 (23.38%)
	> 1 and ≤ 2 cm	333 (42.1%)
	> 2 cm	151(19.09%)
First-line treatment	PDS	633 (80.03%)
	NACT	158(29.97%)

Table S1: Characteristics of HGSOC patients in the study. If the data is continuous, the data represents the number of patients in the group (n) or median with first and third quartiles. The first-line treatment includes neoadjuvant chemotherapy (NACT) and primary debulking surgery (PDS). PFI is a progression-free interval, which is measured from the time at the end of first-line therapy to the date of progression of the disease.

	Aggressiveness line 1	Aggressiveness line 2	Aggressiveness line 3	Aggressiveness line 4
Resistance line 1	**-0.2033**	*-0.0913*	-0.0526	-0.0036
Resistance line 2	-0.0020	**-0.2223**	*-0.1324*	-0.0423
Resistance line 3	*0.1216*	*-0.1658*	**-0.3412**	**-0.2328**
Resistance line 4	0.0220	-0.0502	**-0.2259**	**-0.4242**

Table S2: Summary and correlations of the data-based aggressiveness and data-based resistance measuring from the HGSOC data for the first four lines of therapy. Italics indicate significance at $p < 0.05$ and bold $p < 0.0001$.

The mathematical models result.

We focus on the treatment-sensitive-resistant cells model and the adaptive dynamic model. The single-cell model cannot fit the data as well as the other model, so the analysis for the single-cell model will not be presented. All other three models can be used to fit the dynamic of CA-125 equally well by comparing with the fitting by using the super-smooth function (the result is presented in the main paper, Fig. 4). For this reason, the main paper presents one model, which is the SR and R0 model with *all-lines fitting* and *two-lines fitting.*

We run the survival analysis using Cox analysis after patients finish the second-line chemotherapy. Three models provide similar results of the impact of the parameter estimation: the SR and R0 model (present in the main paper), the SR and μ model (Fig. S2, Tab. S4), and the Adaptive dynamic model (Fig. S3). For each parameter, we divided it into two subgroups, high rate (patients who have a rate of growth or death

greater than the median of that rate) and low rate patients who have a rate of growth or death less than the median of that rate). Patients have longer survival with less model-estimated aggressive treatment-sensitive cells (small γ_S), less model-estimated aggressive in treatment-resistant cells (small γ_R), or lower model-estimated resistance in treatment-resistant cells (larger δ_R), with no effect of model-estimated resistance in treatment-sensitive cells (δ_S).

All-line fitting result

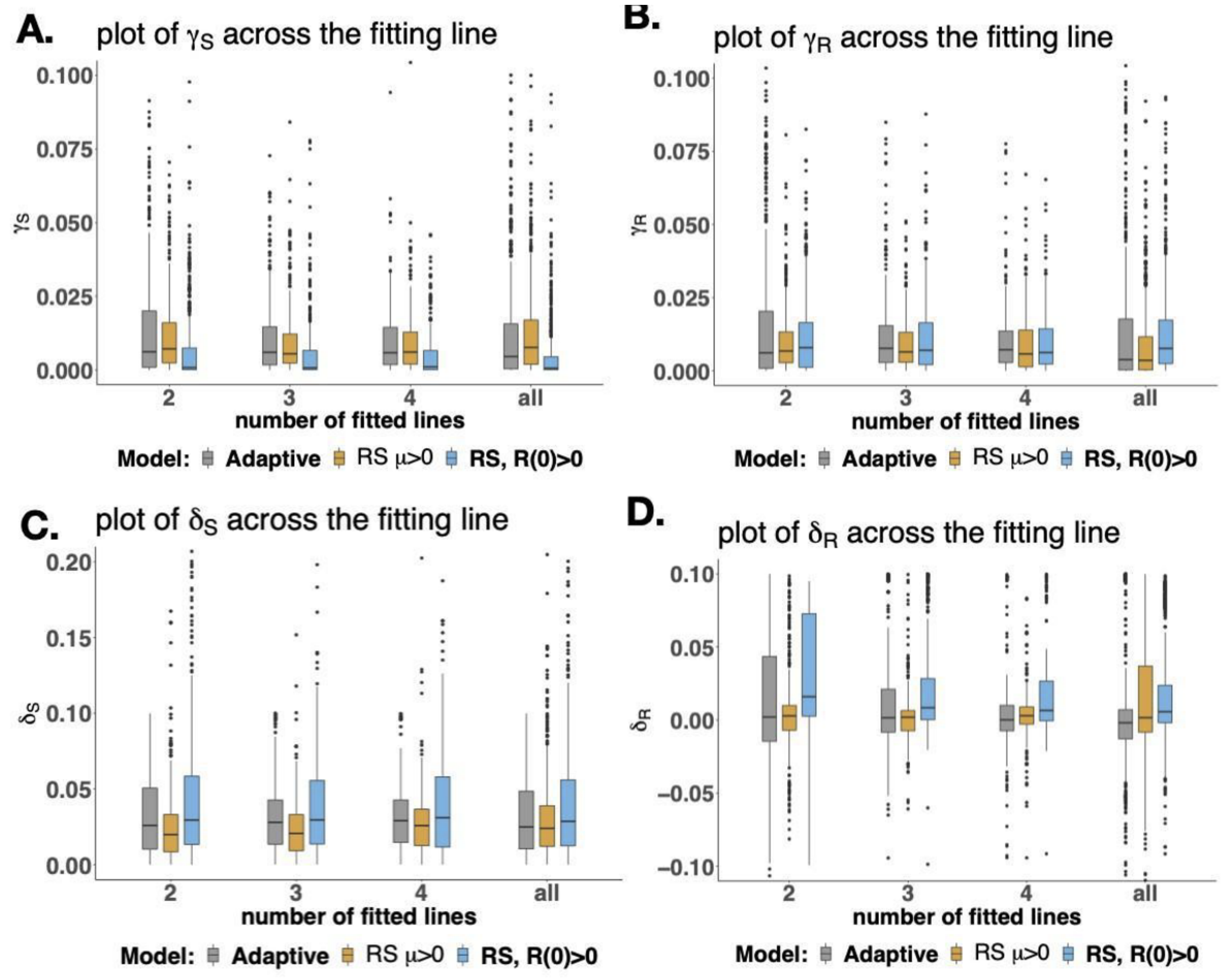

Figure S1: Box plot of parameters estimated from three models: Adaptive dynamic model, the SR and μ model, and the SR and R0 model across the different numbers of fitted lines. **A.** growth rate of the treatment-sensitive cell (γ_S) **B.** growth rate of treatment-resistant cells (γ_R) **C.** death rate of treatment-sensitive cells δ_S and **D.** death rate of treatment-resistant cells (δ_R).

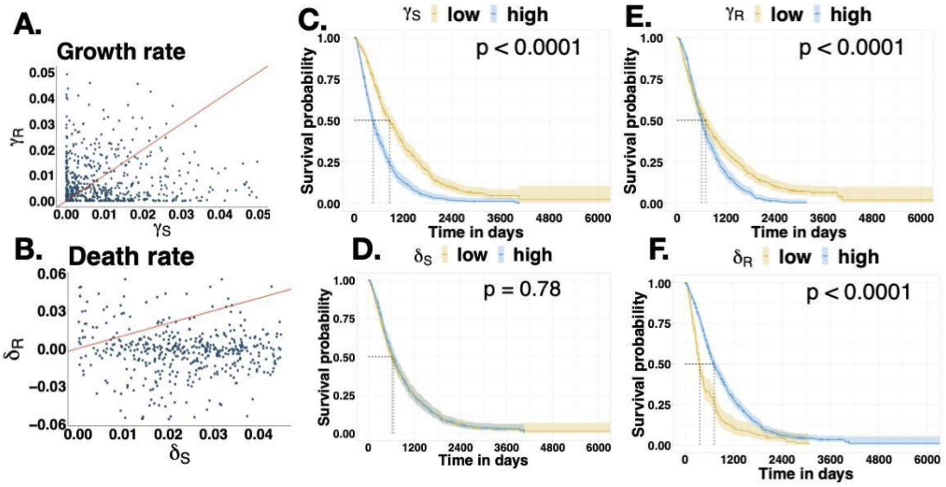

Figure S2: **A.** Model estimated aggressiveness (growth rate) and **B.** model-estimated resistance (death rate) from the SR and μ. The red line is the diagonal where the rates are equal. The KM plot shows the survival probabilities of HGSOC patients with groups based on values of **C.** the growth rate of the treatment-sensitive cell (γ_S), **D.** death rate of treatment-sensitive cells (δ_S), **E.** the growth rate of treatment-resistant cells (γ_R), and **F.** death rate of treatment-resistant cells (δ_R) from the SR and μ model.

Variable	N	Hazard ratio		p
Growth rate of treatment-resistant cells	599		1.76 (1.61, 1.92)	<0.001
Growth rate of treatment-sensitive cells	599		1.14 (1.06, 1.22)	<0.001
Death rate of treatment-resistant cells	599		0.68 (0.61, 0.76)	<0.001
Death rate of treatmement-sensitive cells	599		1.01 (0.92, 1.11)	0.80
Transition rate from treatment-sensitive to treatment-resistant cells	599		1.05 (1.00, 1.11)	0.05

0.8 1 1.2 1.4 1.61.8

Table S3: Forest plot of adjusted hazard ratios (HRs) with 95% confidence interval (CI) of survival time after the second-line treatment by using multivariate Cox regression analysis. The death and growth rates are estimated from all data points fitting from the SR and μ model. N represents the number of patients who were used in the multivariate Cox regression with all variables existing for analysis..

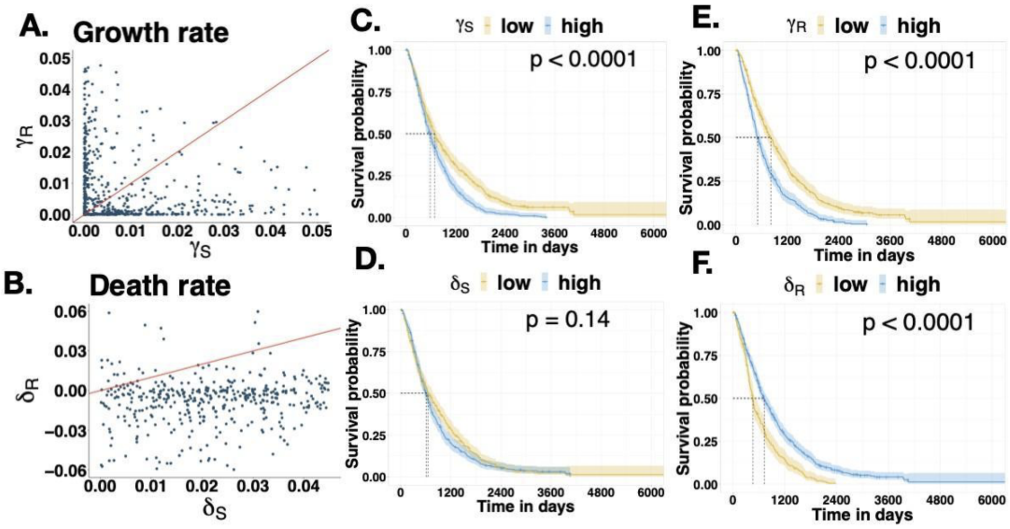

Figure S3: **A.** Model-estimated aggressiveness (growth rate) and **B.** Model-estimated resistance (death rate) from the Adaptive Dynamic model. The red line is the diagonal where the rates are equal. The KM plot shows the survival probabilities of HGSOC patients with groups based on values of **C.** growth rate of the treatment-sensitive cell (γ_S), **D.** death rate of treatment-sensitive cells (δ_S), **E.** the growth rate of treatment-resistant cells (γ_R), and **F.** death rate of treatment-resistant cells (δ_R) from the Adaptive Dynamic model.

Variable	N	Hazard ratio		p
Growth rate of treatment-resistant cells	600		1.60 (1.48, 1.73)	<0.001
Growth rate of treatment-sensitive cells	600		1.25 (1.16, 1.35)	<0.001
Death rate of treatment-resistant cells	600		0.71 (0.64, 0.79)	<0.001
Death rate of treatment-sensitive cells	600		1.12 (1.03, 1.23)	0.01

0.8 1 1.2 1.4 1.6

Table S4: Forest plot of adjusted hazard ratios (HRs) with 95% confidence interval (CI) of survival time after the second-line treatment by using multivariate Cox regression analysis. The death and growth rates are estimated from all data points fitting from the Adaptive dynamic model. N represents the number of patients who were used in the multivariate Cox regression with all variables existing for analysis.

Two-line fitting

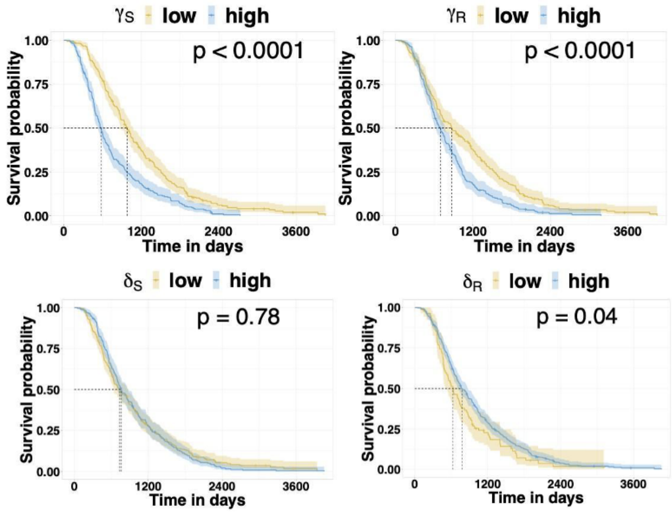

Figure S4: The KM plot shows the survival probabilities of HGSOC patients with groups based on values of **A.** the growth rate of the treatment-sensitive cell (γ_S), **B.** the death rate of treatment-sensitive cells (δ_S), **C.** the growth rate of treatment-resistant cells (γ_R), and **D.** the death rate of treatment-resistant cells (δ_R) from the SR and μ model with two-lines fitting.

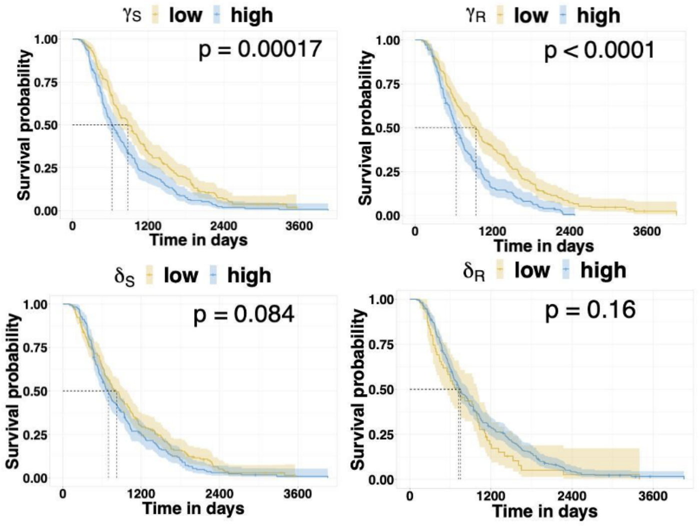

Figure S5: The KM plot showing the survival probabilities of HGSOC patients with groups based on values of **A.** the growth rate of the treatment-sensitive cell (γ_S), **B.** the death rate of treatment-sensitive cells (δ_S), **C.** the growth rate of treatment-resistant cells (γ_R), and **D.** the death rate of treatment-resistant cells (δ_R) from the Adaptive Dynamic model by *two-lines fitting.*

Three-line fitting

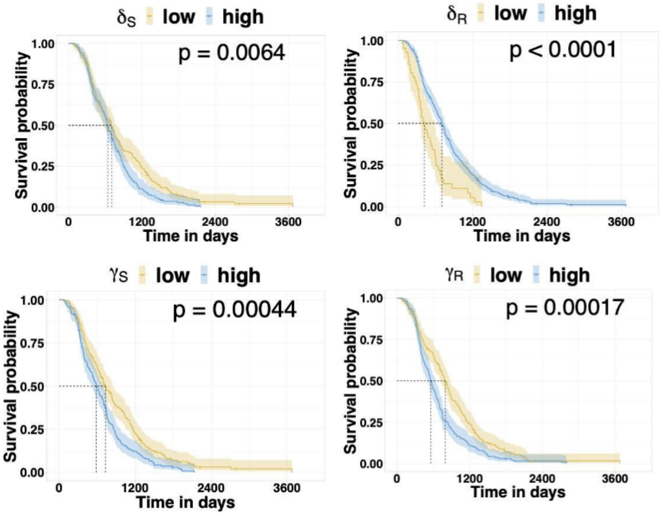

Figure S6: The KM plot shows the survival probabilities of HGSOC patients with groups based on values of **A.** the growth rate of the treatment-sensitive cell (γ_S), **B.** the death rate of treatment-sensitive cells (δ_S), **C.** the growth rate of treatment-resistant cells (γ_R), and **D.** the death rate of treatment-resistant cells (δ_R) from the SR and μ model with *three-lines fitting*.

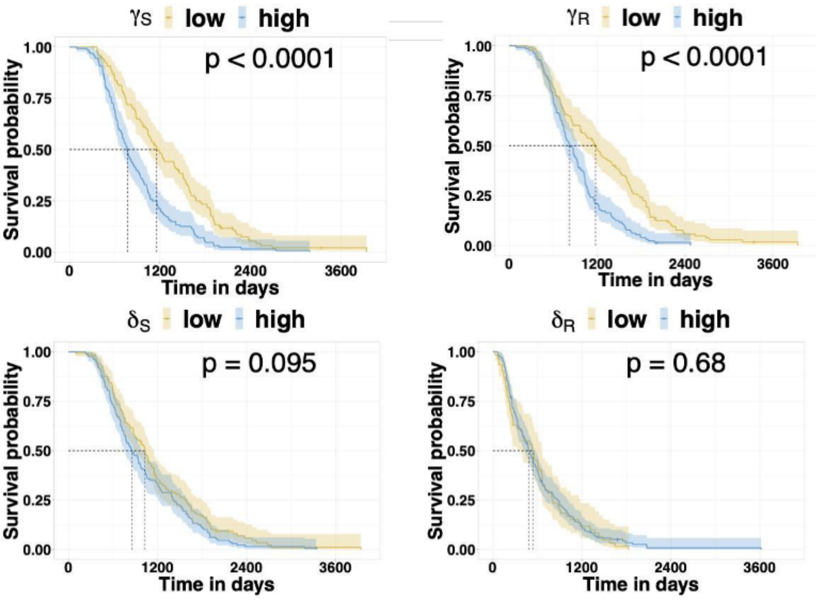

Figure S7: The KM plot showing the survival probabilities of HGSOC patients with groups based on values of **A.** the growth rate of the treatment-sensitive cell (γ_S), **B.** the death rate of treatment-sensitive cells (δ_S), **C.** the growth rate of treatment-resistant cells (γ_R), and **D.** the death rate of treatment-resistant cells (δ_R) from the Adaptive Dynamic model by *three-lines fitting*.

Four-line fitting

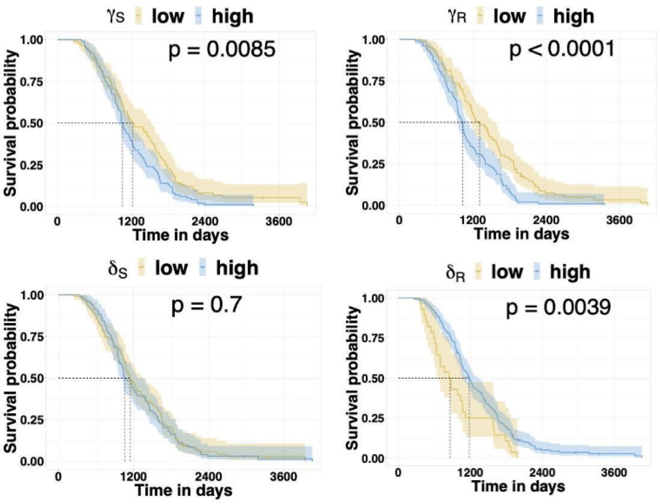

Figure S8: The KM plot shows the survival probabilities of HGSOC patients with groups based on values of **A.** the growth rate of the treatment-sensitive cell (γ_S), **B.** the death rate of treatment-sensitive cells (δ_S), **C.** the growth rate of treatment-resistant cells (γ_R), and **D.** the death rate of treatment-resistant cells (δ_R) from the SR and μ model with *four-lines fitting.*

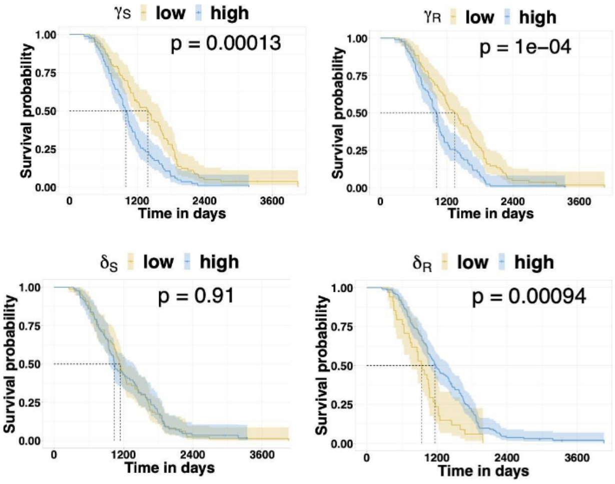

Figure S9: The KM plot showing the survival probabilities of HGSOC patients with groups based on values of **A.** the growth rate of the treatment-sensitive cell (γ_S), **B.** the death rate of treatment-sensitive cells (δ_S), **C.** the growth rate of treatment-resistant cells (γ_R), and **D.** the death rate of treatment-resistant cells (δ_R) from the Adaptive Dynamic model by *four-lines fitting.*
