# Supplementary material for: Mathematical modeling of the evolution of resistance and aggressiveness of high-grade serous ovarian cancer from patient CA-125 time series
Source: PLoS Comput Biol. 2024 May 29;20(5):e1012073. doi: 10.1371/journal.pcbi.1012073 (PMC11164342; doi:10.1371/journal.pcbi.1012073)
Supplement: Attachment — Submitted filename: Jitmana_MathematicalModelingoftheEvolutionofResistanceandAggressivenessofHighGradeSerousOvarianCancerFromPatientCA125TimeSeries_2023_Review.pdf [file pcbi.1012073.s010.pdf]

In this paper, the authors performed a time-series analysis of CA 125 levels in HGSOC to evaluate the development of chemoresistance and response to therapy. They developed and analyzed two mathematical models, focusing primarily on treatment resistance and aggressiveness. The models were calibrated to clinical data from 791 HGSOC patients and model parameters were analyzed to study the evolution of these two traits. The authors aimed to predict overall survival by analyzing CA-125 dynamics during treatment. Model parameters were evaluated for correlations with overall survival. While the study was interesting, there are several areas of concern listed below.

Major concerns

1. For the SR model, it is assumed that the $\delta_R, \delta_S, \gamma_R$, and γ_S are constant over time. Is this a fair assumption given that the treatments change for each line?
2. Figure 2 shows the change in the resistance and aggressiveness between patients for each line. Were these derived from individual lines of therapy of by combining the first and second, first through third, etc.?
3. The patients included in this analysis were required to have undergone second-line treatment after disease progression. Did each patient receive the same therapy during each line? If each patient received a different type of second, third, or fourth-line therapy, the comparison shown in Figure 2 might not be an accurate representation of the population dynamics.
4. What statistical test was used to determine the significance in the Kaplan Meier plots? Specifically, Supplementary Figure S3A. I'm surprised to see a significant difference between the curves considering that they cross and their median TTPs are quite close.
5. Is there a particular reason why the goodness of fit ratio is used in some instances and R^2 is used in others? Please include this in the text.
6. As one of the goals of the paper is to demonstrate that the evolution of resistance and aggressive can predict overall survival, I think it is important to demonstrate that the model can accurately describe the data. On line 209, it says "a ratio close to 1 indicates a good fit." However, the ratios shown in Figure 4 do not demonstrate this. Additionally, line 302 states they are "all significantly less than 1". The medians for three of the four models are around 0.5. The same can be said about the fits shown in Figure S4. Are the models truly able to describe the patient-specific dynamics? This leads me to question the correlations between the parameters and overall survival. If the model parameters do not indeed describe the model dynamics, then specific correlations cannot be established. Perhaps the mechanisms governing each of the equations is wrong.
7. Please include fits to the data for the two-line, three-line, and four-line fitting. From Figure 8, it looks like all the R^2 values are less than 0 for both the next chemotherapy line and all subsequent lines. This again leans to the idea that the models cannot describe the data and the model parameters should be used to assess correlations with overall survival.

Other concerns

1. Please be consistent throughout the text in using "Supplementary" and "Supporting." Several figures and tables were hard to identify due to mislabeling. For instance, line 269

references Supplementary Table S1 Table, however the results described in the text are not reflected in this table.

2. The dashed and solid vertical lines in Figure 1E are not described in the caption. It is not clear if each line indicates a dose of treatment, progression. The gray shading is not described either. Is progression defined via tumor volume or when CA-125 rises above a certain level? This is not clear from this figure or in the text.
3. Overall, the figures could be improved for clarity and consistency. Particularly, Fig. S3, please note the difference in median TTP.
4. The formatting for the tables did not carry over to the manuscript (bold and italics).
5. Please describe Fig 5A before 5B in the text or considering changing the order in the overall figure.
6. Figure 7 is not described in the text.
7. Supplementary Table 7 was not provided.
8. Please consider reordering Figure 1 such that subfigure E is displayed first. Otherwise, please describe A-D before E in the text.
