# Supplementary material for: Mathematical modeling of the evolution of resistance and aggressiveness of high-grade serous ovarian cancer from patient CA-125 time series
Source: PLoS Comput Biol. 2024 May 29;20(5):e1012073. doi: 10.1371/journal.pcbi.1012073 (PMC11164342; doi:10.1371/journal.pcbi.1012073)
Supplement: Attachment — Submitted filename: Reviewer response.pdf [file pcbi.1012073.s011.pdf]

Reviewer comment:

Reviewer 1:

In this paper, the authors performed a time-series analysis of CA 125 levels in HGSOC to evaluate the development of chemoresistance and response to therapy. They developed and analyzed two mathematical models, focusing primarily on treatment resistance and aggressiveness. The models were calibrated to clinical data from 791 HGSOC patients and model parameters were analyzed to study the evolution of these two traits. The authors aimed to predict overall survival by analyzing CA-125 dynamics during treatment. Model parameters were evaluated for correlation with overall survival. While the study was interesting, there are several areas of concern listed below.

Main concern:

1. For the SR model, it is assumed that the delta and gamma are constant over time. Is this a fair assumption given that the treatments change for each line?

   Author: In the SR model, the effective growth and death rates change due to the frequency of the two cell types. By allowing delta and gamma to change with two cell types, they have many more parameters than either the SR model or the Adaptive Dynamics model. In our recent research, we aim to use the most basic model to analyze the CA-125 level, as its simplicity allows for easier interpretation, fewer assumptions, and often a more robust performance with less parameters. By fitting it to the data, we can efficiently identify trends and patterns without the complexity of models.

2. Figure 2 shows the change in the resistance and aggressiveness between patients for each line. Were these derived from individual lines of therapy of by combining the first and second, first through third, etc.?

   Author: We apologize for the lack of clarity. The figure shows the response during each distinct treatment line. This approach assesses the dynamics of resistance and aggressiveness as separate and distinct measures for each patient. Additional explanations and examples of the data-based resistance and aggressiveness have been added to Figure 1A.

3. The patients included in this analysis were required to have undergone second-line treatment after disease progression. Did each patient receive the same therapy during each line? If each patient received a different type of second, third, or fourth-line therapy, the comparison shown in Figure 2 might not be an accurate representation of the population dynamics.

   Author: In the clinical management of patients, the choice of second-line treatment is typically determined by a combination of factors, including the response to the first-line therapy and consideration of potential side effects. This decision-making process often results in patients receiving various types of second-, third-, and fourth-line therapies.

The primary objective of this paper is to evaluate a simplified scenario that does not consider different treatment types for the sake of analytical clarity and statistical power. This simplifying assumption effectively assumes strong cross-resistance among treatments. Exploration of models incorporating different drug types is a focus of ongoing research efforts by the authors.

4. What statistical test was used to determine the significance of the Kaplan Meier plots? Specifically, Supplementary Figure S3A. I'm surprised to see a significant difference between the curves considering that they cross and their median TTPs are quite close.

   Author: The Kaplan-Meier plots are analyzed for significance using the log-rank test. In this study, we used the Wilcoxon test to the survival analysis and computed the p-value.

5. Is there a particular reason why the goodness of fit ratio is used in some instances, and R2 is used in others? Please include this in the text.

   Author: We use the goodness of fit ratio to assess fits to observed data because it is a more rigorous evaluating of the fit than R^2. When predicting future data, we use R^2 to illustrate the predictive ability of the model to CA-125 level in the short and long run because it is a well-known model fitting evaluation method. More detail of the explanation can be found in the Model comparison session in the line 220-241 of the main text.

6. As one of the goals of the paper is to demonstrate that the evolution of resistance and aggressiveness can predict overall survival, I think it is important to demonstrate that the model can accurately describe the data. On line 209, it says, "a ratio close to 1 indicates a good fit." However, the ratios shown in Figure 4 do not demonstrate this. Additionally, line 302 states they are "all significantly less than 1". The medians for three of the four models are around 0.5. The same can be said about the fits shown in Figure S4. Are the models truly able to describe the patient-specific dynamics? This leads me to question the correlation between the parameters and overall survival. If the model parameters do not indeed describe the model dynamics, then specific correlations cannot be established. Perhaps the mechanisms governing each of the equations is wrong.

   Author: The value of 0.5, as presented in the model fitting section, is in comparison with Friedman's SuperSmoother function fit, which effectively uses all of the data and is a rather good fit, particularly given the simplicity of the models. The power of the estimated parameters to predict survivorship is an independent test of the value of the models, and we argue that the models are sufficiently good to capture critical differences among patients.

7. Please include fits to the data for the two-line, three-line, and four-line fitting. From Figure 8, it looks like all the R2 values are less than 0 for both the next chemotherapy line and all subsequent lines. This again leans to the idea that the models cannot describe the data and the model parameters should be used to assess correlations with overall survival.

Author: We have included the information of the fitted line of two, three, and four lines fitting to Figure 8A. According to Figure 8A, the R^2 for the data used in the fitting has a median of 0.7, which is a good indicator that the model can fit the data well. Nonetheless, it is true that this model fails to predict the subsequent lines of treatment by using some of the initial time course, as shown in the negative R^2 in Figures 8B and 8C.

Other concern:

1. Please be consistent throughout the text in using "Supplementary" and "Supporting." Several figures and tables were hard to identify due to mislabeling. For instance, line 269 references Supplementary Table S1 Table, however, the results described in the text are not reflected in this table.
   All of the text is consistent by using Supporting information.

2. The dashed and solid vertical lines in Figure 1E are not described in the caption. It is not clear if each line indicates a dose of treatment, progression. The gray shading is not described either. Is progression defined via tumor volume or when CA-125 rises above a certain level? This is not clear from this figure or in the text.
   The individual vertical line denotes the date the patient received the treatment, which we called "Cycle of treatment". The varying colors of the vertical lines indicate the use of distinct drugs in each treatment regimen. The group of cycles together with the gap between cycle is called, "lines of treatment". The first line, second line, third line, fourth line, and fifth line indicate the line of treatment, during which alpha = 1. All the above explanation is added to Figure 1A, we rearrange figure 1 to be consistent with the order presenting in the main text.

3. Overall, the figures could be improved for clarity and consistency. Particularly, Fig. S3, please note the difference in median TTP.
   The median difference has been added to Figure S3.

4. The formatting for the tables did not carry over to the manuscript (bold and italics).
   The bold and italics are fixed for all tables.

5. Please describe Fig 5A before 5B in the text or considering changing the order in the overall figure.
   The text is now in the correct order by describing Figure 5A before 5B.

6. Figure 7 is not described in the text.
   Figure 7 is added to the text.

7. Supplementary Table 7 was not provided.
   I am sorry for this typo. The supplementary Table 7 has been removed from the text.

8. Please consider reordering Figure 1 such that subfigure E is displayed first. Otherwise, please describe A-D before E in the text.
   Figure 1 is now re-ordered to correspond with the order presented in the text.

Reviewer 2:

1. I can't find the units of growth/death rate – have these been reported? I would appreciate a table of parameters (units, values, brief description) for the ODE models.
   Author: The summary of parameters has been added to the caption under Table 1. The value range is shown in the results because it is based on the model fitting and differs across patients. Table 1 describes each parameter used in the SR and AD models with their units, and all rates are "per day."

2. Authors appear to assume exponential growth in both ODE models (e.q's 3-5), could authors justify this choice or else check results are not affected by using another model (e.g. logistic). As a general point, it is not clear whether the authors have used existing models (i.e. from citations 32,35,36,38), whether existing models have been modified, or if the models presented here are entirely new. I would like to see more explicit comparison of similarities/differences with existing models and justification of any differences.
   Author: We have added explanations of previous models to the introduction in Lines 69-75. The model presented in this paper is not intended to be new but chosen as the simplest model, which we modified from the models of the biomarker PSA in prostate cancer. We did not perform model comparisons with more complex pre-existing models, many of which were developed for PSA, which has different dynamics and treatments than ovarian cancer. This is because our statistical analysis with linear and nonlinear regression found that the exponential model best fits the dataset during both the growth and decline phases.

3. Both AD and SR models assume zero tumor growth during treatment, which seems unrealistic (especially for resistant cells). Indeed in Fig. S4B it does look like tumors progress during treatment intervals. Given the large data set, it should be possible to estimate the parameter alpha directly from the data.
   Author: We apologize for the lack of clarity on the parameter alpha. It is an indicator variable that is directly derived from the dataset, with alpha = 1 during each treatment line, including first-line therapy, second-line therapy, third-line therapy, and so on. When treatment is not given alpha=0. Examples of alpha values are shown in Figure 1A. We have also clarified that the current model does allow the resistant cells to grow during the treatment, as the reviewer notes presented in lines 151-152. During parameter estimation, we allow delta_R to be both positive and negative, with negative values of death rate indicating the growth of resistant cells during treatment.

4. I can find no legend/description of the vertical colored bars in Figs 1, S2, & S4.
   Author: The descriptions have been added to each Figure. The individual vertical lines indicate the dates on which the patient received the treatment doses, which is called the cycle of treatment. The group of Cycle together with the gap between cycles is called Line of therapy. The varying colors of the vertical lines indicate the use of distinct drugs in each treatment regimen. The first line, second line, third line, fourth line, and fifth line indicate the line of treatment.

5. I would like to see some discussion about what is considered to be "on treatment" in the model. As an example, in Fig. S4B there are vertical blue bars, which I presume represent treatment intervals. In the model, is it assumed that patients are "off treatment" during the small intervals between these blue bars, or is the patient considered to be "on treatment" throughout? What about S4B yellow bars, where the time intervals are much longer?
Author: We apologize for the lack of clear definitions. In accordance with standard clinical practice, we consider patients to be "on treatment" during the entire period between the first and last vertical bars (cycles of treatment) of each line, even when the gaps between doses (cycles) are significant. Thus, the alpha value, as described in Figure 1A, is set to 1 during the entire interval to indicate the on treatment while it is 0 to indicate the off treatment.

6. I am not convinced about the robustness of the authors parameter estimation procedure. E.g. Fig. S4C single cell model fit looks very poor, it seems the data could be fit much better with a horizontal line (which the model structure does not preclude). Evidence that authors had taken steps to find global optima during parameter estimation (e.g. by performing repeated fits with different initial parameter guesses) would help assuage these doubts.
Author: The model fitting process was started from a common initial guesses for all parameters in all patients which was derived from the data, referred to as data-based aggressiveness or resistance within the paper. The data-based aggressiveness and resistance for the first line is used as the initial guess for gamma_S and delta_S, repectively. On the other hand, the median for data-based aggressiveness and resistance for the remaining lines are used as initial guess for gamma_R and delata_R, respectively. We use the same initial guess across all models, allowing them to vary across individual patients. The horizontal line does not fit better in the current model because of the rapid drop of the CA-125 during the first line of treatment. The model output with all parameters set to 0 would remain at the initial condition and provide a poor fit.

7. On line 312 authors write "There was no relationship between the estimated growth or death rates of sensitive and resistant cells". This would be an interesting finding since the presence of a fitness cost of resistance is an open question with therapeutic relevance (see e.g. Gatenby & Brown, 2018). Moreover it does look like there might be a negative correlation in Fig. 5A. Authors should state which correlation coefficient they have used – would different coefficients give different results?
Author: The correlation coefficient is shown in Table 3. We used the Pearson correlation though out the study to test the linear correlation between the parameters. Specifically, the correlation coefficient for the estimated growth of sensitive cells (γ_R) and resistant cells (γ_S) and the death rates of sensitive cells (δ_S) and resistant cells (δ_R) are calculated. A positive correlation is found between the growth rate of sensitive cells (γS) and their death rate (δ_S), and a similar positive correlation is observed between the growth rate of resistant cells (γ_R) and their death rate (δ_R). However, when comparing the death rate of sensitive cells (δ_S) with the death rate of resistant cells (δ_R), and the growth rate of

resistant cells (γ_R) with the growth rate of sensitive cells (γ_S), no correlation is evident.

8. Authors model selection approach is non-standard & doesn't take into account the number of estimated parameters. Why not use e.g. AIC/BIC? Moreover it is not clear why one particular model variant was chosen for Figs 6-8 and the others are relegated to the supplement.
Author: In the present study, our primary objective is to assess and compare the predictive performance of each mathematical model rather than focusing on selecting the most appropriate model. We present only the outcomes of the model incorporating initial resistant cells for brevity because there were no significant differences among the models considered as shown in Figure 4 in the main text.

9. In the supplementary file a figure reference appears to be missing "(the result is presented in the paper, Fig. ??)". The figure has been added.

10. Authors concede in the discussion that their model neglects differences between different types of therapeutic agents. It would be useful to have more information about what treatments patients received (a Sankey diagram might be a good visual representation of this). If there are some treatment combinations with enough patients, it would be interesting to see how the accuracy of model predictions for future response depends on the treatment combination.
Author: Thank you for making this point. As noted in our response to reviewer 1, the primary objective of this paper is to evaluate a simplified scenario that does not distinguish among treatment types for the sake of analytical clarity and statistical power. Exploration of models incorporating different drug types is a focus of our ongoing research.

Reviewer 3:

In this work, the authors evaluate and investigate chemoresistance in ovarian cancer patients and predict treatment effectiveness based on individual characteristics analysed from the data (using a mathematical model of log-slope).
Cancer Antigen 125 (CA-125) levels were measured in 791 patient with high-grade serous ovarian cancer (HGSOC). They evaluated the effectiveness of different mathematical models of treatment sensitive and treatment resistant cancer cells (ODEs, AD) and fit to the HGSOC data and captured the CA-125 level. They Found that patients with log growth rates of treatment-sensitive cells and treatment-resistant cells and a high death rate of treatment resistant cells have longer survival
I have a few comments below around clarity of some explanations and suggestions for delving further into the data/model predictions. Overall, I think this is an interesting study.

Major concern:

1. The authors mention that they calculate the "data-based aggressiveness as the slope of the log-transformed CA-125 level changing when patients were not receiving therapy"
1.1) can the authors comment on how this (and the data-based resistance measure) were

confirmed to be a good measure to quantify the data?
Author: The slope characterizes the rate at which the CA-125 level changes when patients are between lines treatment. Consequently, the values of resistance and aggressiveness serve as descriptors how patients respond to during and between treatments as mention in line 112-120 in the main text.The example of data-based-aggressiveness and data-based resistance for each line of treatment has been added to Figure 1A for clarification.

1.2) In between treatment lines it seems for the data shown that there is a decrease then increase, how is this captured in the single measurement? If the slope changes between the lines of treatment, how is this captured?
Author: We investigated models where the treatment effect continues after the final dose, which can create a delay before CA-125 begins to increase. These models did not provide better fits to the data despite their additional complexity. For clarity, each estimate is done separately for every interval on and off treatment.

1.3) I'd be curious to see what other measures might provide more or different information than what is captured in Fig 2. It seems there are very long tails for the predicted slopes, why is this?
Author: The long tails, these are due primarily to the variation in the number of data points per patient. Patients with only two data points for calculating the slope introduce outliers in the estimates.

2. Can the authors comment on the reasons for delta_R not constrained to being positive? In theory it means when the drug is on, we can have growth of resistant cells be represented by this death term. I think some comment around this is necessary for the reader.
Author: Thanks for this suggestion, and we have added further clarification to the model description under the model assumption for parameters, which can be found in lines 154-155. Indeed, our central idea is that resistant cells can withstand the effects of treatment and grow during treatment. Because the models are linear, it is appropriate to treat growth as negative death (which is not true for the logistic model).

3. In the data in Fig 1E, it is clear there can be a decline after treatment line ends and then eventually an increase. In the model, treatment on or off is controlled by alpha = 0 or 1. This means when the treatment line ends that alpha = 0 and we should only have growth of both population (as the delta terms should be zero) but the data doesn't seem to depict this, can the authors explain whether this means Eq (3) and (4) don't match data that looks like Fig 2? Or what might be causing a decline after treatment line ends that is captured in their model.

Author: The post-treatment decline in CA-125 levels can be attributed to several factors, including residual drug persistence in the patient's body, owing to the drug's extended half-life. To explore this scenario, we conducted investigations by incorporating delays in the drug response, simulating the persistence of 14 days, 30 days, and 45 days after the treatment lines had concluded, as shown in the figure below. Our modeling experiments revealed that these delays had no discernible impact on the model's ability to fit the data effectively. Based on these findings, we present results from the simpler model without such a delay.

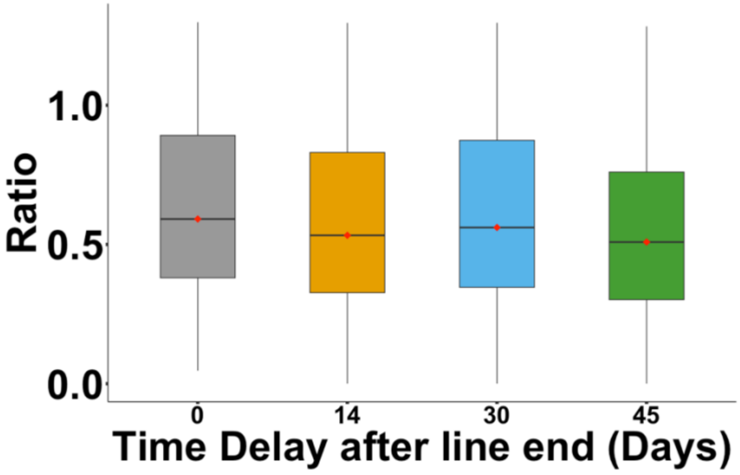

Figure: The boxplot shows the goodness of fit ratio of the SR model with initial value of resistant cells, which compare the model fitting to the fitting using the SuperSmoother function. The x-axis indicates the time of the delay after the end of the lines if we allow the CA-125 level to be able to decline after the lines have ended.

4. I think the correlations across patients is very interesting and could be dug into further and moved into the main text (Table S1). e.g.
4.1) does the minimum CA-125 between lines correlate to longer term survival?
Author: There exist no significant relationship between the minimum value of CA-125 level in the treatment lines to the survival probability.

4.2) if you only estimate the slope post minimum between lines, does this provide a better indicator of resistance?
Author: We performed piecewise linear regression on the data set starting from line one to the starting of line two to estimate the rate of change of CA-125 decreasing and increasing in this time course. It provided a better survival prediction than resistance and aggressiveness measured separately. As we mentioned above, adding the delay after the end of the line does not improve the model fit. For this reason, we decided to exclude the result in the paper.

4.3) Does aggressiveness and resistance at each line correlate for individual patients?
Author: A correlation exists between data-based aggressiveness and data-based resistance presented in Supplementary Table 2. The correlation between model-estimated aggressiveness and resistance is presented in Table 2.

4.4) Is there a correlation between parameter values or data values and the style of treatment (length of line? Or treatment type?)
Author: The data show no correlation between treatment duration and parameter values. We have tested the treatment type of line 1 between PDS and NACT, which also shows no difference in parameter range. As noted earlier, we are not considering different treatment types in this study to focus on the simplest possible models.

5. It would be interesting to see whether of the "three general dynamical patterns" one model fits one better, instead of just looking at all data fits per model today (Fig 4)
   Author: Thank you so much for the suggestion. We are currently working to find a rigorous way to cluster patients and include these solely as illustrations of three general dynamic patterns.

Minor.

1. I'm not sure if this is a typo but I'm unfamiliar with the name of a study being in the author list: "Australian Ovarian Cancer Study", should that be in the author list?
   Author: The Australian Ovarian Cancer Study group requested this recognition of the work by the large team of people who recruited the patients and assembled the data used in this analysis.

2. The introduction feels lacking in references to previous mathematical works modelling ovarian cancer resistance. I encourage the authors to make a small addition to the introduction mentioning previous models of Ovarian cancer treatment or of cancer resistance modelling. It allows the readers to contextualise this work and understand it's advantages over previous models that may be available. I see they add some references, but the discussion around these and the breadth of these references feels lacking.
   Author: We have added explanations of previous models to the introduction in Lines 70-75. The model presented in this paper isn't entirely new but modified from simple models of the PSA biomarker in prostate cancer. To our knowledge, there are no existing models of resistant and sensitive cells model or adaptive dynamics for ovarian cancer.

3. The figures are quite pixelated in the version I'm seeing – could this be fixed for publication:
   The figures have been improved in quality.
4. Can more information be given on the treatments, the authors mention "first-line", "second-line" etc but it's not explained what this means, in particular:
   4.1) Was each first, second etc line treatment the same for each patient
   Author: Sorry for the lack of clarity. The Figure representing each line is shown in Figure 1A. The first line of systemic therapy is similar for most ovarian cancer patients, however the choice of subsequent lines of treatment for relapsed disease can vary and depends on multiple factors such as the response to the previous line, the side effects of drugs, patient age, etc.

   4.2) How long are these line treatments? E.g. one a day for 7 days? How heterogeneous is this across the cohort of patients?
   This varies depending on the type of systemic treatment. The number of cycles per line and the time interval between cycles differ depending on the therapy regimen. Line of treatment (including maintenance therapy) ranged from 0 to 2702 days with a median of 106 days. The time between treatment lines ranged from 0 to 4879 days with a median of 165 days.

5. Can the authors explain their mixed-effects linear regression fitting process in more detail? They reference using R, can the code please be provided (even for a synthetic dataset). They explain a least-squares fitting more in detail but mention the mixed-effects model earlier.

Author: The description is added to the lines 121 to 127. The fixed effects are used to model constant factors of interest, while random effects capture sources of variability that are not the primary focus of the analysis. Fixed effects are typically predetermined by using individual data, while random effects are estimated from the whole group of data sets. We used standard syntax in R with lm and lme4 for these regressions.

6. Typo in Fig S4 caption "/mu" The typo has been fixed.

7. Can the corresponding pair-parameter plots to Fig 5A and B be given to see what the other pair correlations are (if any) The new description in a paragraph has been matched with the order of figure.

8. How was c1 and c2 in (1) estimated? How do they feed into the model? There exist no study showing the relationship between the CA-125 level to the treatment-resistant or sensitive cells. For this reason, we cannot estimate these parameter from the data. We assumed them to be 1 in the current study. We now removed them from the main text to avoid the confusion to the reader.

9. I find the reference to the three types of models a bit confusing, while there is a paragraph explaining it on the bottom of Pg 17 it would be helpful to have a diagram or shorter names. In addition, the caption to Fig 4 should explain the meaning of the horizontal axis model names again for the reader.
   The explanation of the horizontal axis has been added to the figure.

10. Sometimes the authors say "Supplementary Fig. 4" and sometimes just S4, I'd be consistent throughout
    The reference is consistent though out the paper by using supporting information.
