# Supplementary material for: Mathematical modeling of the evolution of resistance and aggressiveness of high-grade serous ovarian cancer from patient CA-125 time series
Source: PLoS Comput Biol. 2024 May 29;20(5):e1012073. doi: 10.1371/journal.pcbi.1012073 (PMC11164342; doi:10.1371/journal.pcbi.1012073)
Supplement: Attachment — Submitted filename: Reviewer_comment_edit2.pdf [file pcbi.1012073.s012.pdf]

Reviewer's Responses to Questions
Comments to the Authors:
Please note here if the review is uploaded as an attachment.
Reviewer #1: Thank you for your responses to my concerns. While the paper has significantly improved, there remain issues regarding the robustness of the model fitting process.

Specifically, there are conflicting points in the text regarding the model fits to the data. Lines 239-240 state that when the “ratio approaches 1, the model aligns closely with the observed data.” However, the ratios shown in Figure 4 show that they are closer to 0.5, meaning that the RSS_model is higher than the RSS_sup. In fact, lines 344-345 state that the ratios (for all but the single state model) are “all significantly less than 1,” which would imply that the models aren’t able to describe the data. The fits shown in Figure S4 also lend to this point, especially in the long term in panel B. Panel C of Figure S4 also show that the SR and R0 and adaptive dynamics models cannot describe these types of CA-125 dynamics, as their curves are more or less constant despite CA-125 changing throughout the treatment cycles.

Author: An RSS ratio below 1 shows that the model fit is not as good as the super smoother, but this does not necessarily indicate a poor fit. The super smoother fits to all of the data, while the dynamical model uses only the initial conditions and a small number of parameters, meaning that a ratio of 0.5 is rather good. As recommended, we include R^2 and AIC to assess the fit and make formal model comparisons directly. Figure S4C appeared as a straight line due to axis scaling, and we have corrected it to reflect model dynamics accurately.

Additionally, from the distributions of the model-estimated aggressiveness and resistance parameters shown in Figure 5, it appears that the aggressiveness and/or resistance parameters are 0 for many patients. This implies that the optimizer did not converge and instead went to the lower bound. That is likely the case for the fits shown in Figure S4C. If both aggressiveness and resistance rates are 0, then CA-125 might initially drop during the first treatment cycle, but then remain constant once treatment is turned off (which is what we’re seeing in Figure S4C). This lends to the idea that the model is not appropriate for these types of patient data. A global optimization needs to be done to ensure that the models are not collapsing at their bounds.

Author: We tweak the parameters slightly during the fitting process to prevent them from getting stuck at their limits. We've observed no cases where a patient's estimated rates of both aggressiveness and resistance were zero. However, because the values for gamma were plotted on a scale ranging from 10^{-5} to 10^{-1}, with a median of 10^{-3}, most values appeared near zero. To address this and provide a clearer view, we show the gamma values on a logarithmic scale, as shown in the revised graph below. This plot is presented in Supplementary Figure 5.

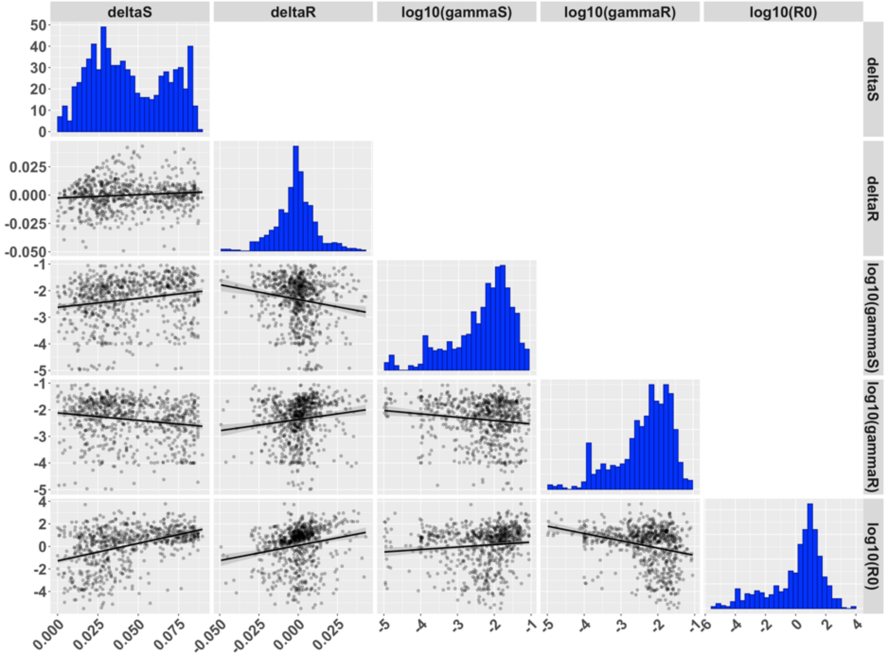

Finally, please include the goodness of fit values (or R^2) for each model for the fits shown in Fig S4. (And maybe even consider including different patients, as the current fits do not describe the data very well.) The R^2 values shown in Figure 7A for 4 lines of therapy are more convincing that the models can accurately describe the data. I'd be curious to see the R^2 values over all lines of the data.
Author: The information has been added to figures and text.

Reviewer #2: Modifications in the revised manuscript have increased clarity and improved the manuscript overall. However, my concerns about the parameter estimation process and protocol for model comparison were not addressed. Since the estimated parameters values are important for the conclusions of the paper, steps should be taken to demonstrate the robustness of the parameter estimates.

"The model output with all parameters set to 0 would remain at the initial condition and provide a poor fit."
This response highlights a flaw in the fitting procedure, namely that the initial level of CA-125 was apparently not included as a parameter to be estimated. Instead all fits seem to be constrained to pass through the first data point, which has the undesirable effect of overstating the importance of the first data point. The impact of this constraint on the results can most clearly be seen in figure S4B, where the gradient of the line is much steeper than it would be had the fit been unconstrained at the first timepoint.
I struggle to believe the claim (regarding the yellow line in figure S4B) that "the horizontal line does not fit better in the current model", because the yellow line is simply so far from the data points. It could be the case that the fit appears worse than it actually is, because the fitting was performed on non log-transformed data whilst the plot displays log-transformed data (which if true, should be indicated on the plot y axis or in the legend). However even then I have difficulty imagining that a horizontal line would provide a worse fit, particularly if the initial condition is fitted as a parameter as it ought to be.
Author: Thank you so much for pointing this out. We've clarified our paper to better explain how we estimate our model's starting conditions. Initially, we take the CA-125 level as a known starting point, not an estimated one. However, we estimate the initial number of resistant cells for the SR, R0 model. Because we consider CA-125 (C) to be the sum of resistant (R) and sensitive (S) cells, the initial CA-125 value is set as $C_0=R_0+S_0$. Consequently, the initial number of sensitive cells is calculated by subtracting the number of resistant cells from the total value of CA-125. The current model operates under a fixed initial condition, C(0). We tested the suggestion to treat the initial condition as an additional parameter, and found that it has a relatively small impact on the model fit and does not change the overall results (see SI5 Fig).
In addition, we updated Figure S4B to include the R^2 value. This shows a value of 0.3596 for single cells, indicating that our model aligns better with the patient data than a simple average of all CA-125 levels would suggest.

"In the present study, our primary objective is to assess and compare the predictive performance of each mathematical model rather than focusing on selecting the most appropriate model."
Even if the goal is to compare but not select a model, common model selection criteria (AIC, cross validation) are a widely used and accepted basis for comparing models. Two criticisms of the current method of comparison are 1) does not take into account model complexity, and 2) uses the super smoother as a benchmark for model performance. The super smoother is itself a nonparametric model of the data, so it seems very strange to benchmark the parametric models against this other nonparametric model rather than simply against the data itself. If there was some compelling reason to use this approach rather than standard criteria, I cannot find it in the text. It would be helpful if the authors could point to instances in the literature where this approach has been used for model comparison before. This approach should be much better motivated or else replaced with a more suitable alternative.
Author: It is an excellent point to provide the more well-known comparison criteria. We calculated each model's AIC and R^2 values from fitting mathematical models using *all-line fitting*, as shown in Figure 4. As both methods are measured for model comparison, we found that all three models (rather than the single-cell model) do equally well in data fitting.

Reviewer #3: Thank you for your revision of your manuscript. I have four remaining comments.

1. In my previous review (Major 1.2 and 1.3), I commented on the miss-match between the data and the model after the first and second treatment line. The authors said they attempted to fit the changing slope using a fixed delay and it made no difference to their ability to fit the model, however, this still concerns me as there is a clear decrease post first and second line treatment in Fig S2 and Fig 1A that contrasts with what is modelled. Potentially, this isn't common across the patients, and maybe that should be included in the text. Either way, I think a more detailed explanation in the text is needed about why this isn't important and the results of attempting to change the model to match it for clarity for the audience. If the details have already been added, can the line numbers be pointed out.

Author: We calculate each patient's delay date by identifying the point at which their data begins to rise after a decline, using piecewise regression analysis using package *Segment* in R on each line. We then determine the time difference between this turning point and the line's endpoint. The table below shows these differences for the first five treatment lines,

Line	1st Qu	median	mean	3rd Qu.
1	-33.80	15.82	77.11	78.07
2	-48.65	-8.11	30.73	2646.72
3	-50.25	-13.21	-10.70	36.84
4	-49.794	-18.853	-5.506	16.215
5	-43.50	-18.64	-18.13	13.69

This shows that, on average, the turning point happens before the end of the line. This might explain why adding the delay after the line of treatment makes no significant difference in model fitting. Moreover, as mentioned in the Introduction, the change in CA-125 can be impacted by other factors such as menstruation, endometriosis, pregnancy, pelvic inflammatory disease, liver disease, benign ovarian cysts, etc. Because we excluded all other factors impacting CA-125 and due to the need for improvement in model fitting by adding a delay after the end of the line, we decided not to include this in the main paper.

Have the authors examined fixing the delay to be the turning point in the patient data? I'd be interested into why this wouldn't give a better fit to the data.
Author: The following figure shows the results with a fixed delay in the turning point after each line of treatment. We can see no significant difference from the model without delay. As stated above, not all patients show any delay after the end of a therapy line, perhaps explaining the lack of an improved fit to the data.

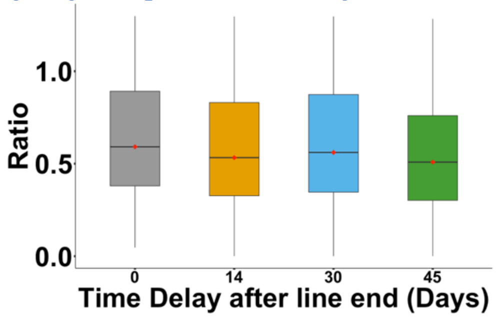

2. Also, can the authors please add the details in their responses to my questions in the round 1 revision to the text. In particular,
• Comment Major 4 – can the authors add to the text their analysis of the correlations between variables and significance/non-significance of these relationships
Author: This information is presented in supplementary tables SI1 and SI2. The text information is given on lines 312-321 in the text.
• Comment Minor 4 – give more details of the range seen for timing and schedule of dose lines across patients
Author: This information is added to lines 328-331
• Comment Minor 5 – add “R with lm and lme4” for transparency and reproducibility.
Author: This is added to the text lines 120-123

3. I do not understand the authors answer to Minor 7, can they please provide either further explanation of their response or re-examine my question. Pointing out line numbers would also help.
Author: Sorry for the misunderstanding in the previous response; it was poorly explained. To avoid multiple plots for parameter combinations to show parameter correlation, we presented them as a correlation table in Table 3 of the main text and the correlation table of the three models with scatter plots, distribution, and correlation coefficient in Supplementary Figure SI5. For clarification, we have added plots for all parameter combinations in Supplementary Figure SI5.

4. Having a Fig S2 and a Fig S2 in the SI needs to be clarified. Please change the names of the figures in the SI to something else, e.g., Fig SI2.
Author: All supplementary information files, figures, and tables have been changed to SI.
